# Utilizing machine learning for early screening of thyroid nodules: a dual-center cross-sectional study in China

**DOI:** 10.3389/fendo.2024.1385167

**Published:** 2024-06-14

**Authors:** Shuwei Weng, Chen Ding, Die Hu, Jin Chen, Yang Liu, Wenwu Liu, Yang Chen, Xin Guo, Chenghui Cao, Yuting Yi, Yanyi Yang, Daoquan Peng

**Affiliations:** ^1^ Department of Cardiology, The Second Xiangya Hospital, Central South University, Changsha, Hunan, China; ^2^ Research Institute of Blood Lipid and Atherosclerosis, Changsha, Hunan, China; ^3^ Department of Cardiology, The Fourth Affiliated Hospital of Soochow University, Suzhou Dushu Lake Hospital, Medical Center of Soochow University, Suzhou, Jiangsu, China; ^4^ Department of Nephrology, The Key Laboratory for the Prevention and Treatment of Chronic Kidney Disease of Chongqing, Chongqing Clinical Research Center of Kidney and Urology Diseases, Xinqiao Hospital, Army Medical University (Third Military Medical University), Chongqing, China; ^5^ Health Management Center, The Second Xiangya Hospital, Central South University, Changsha, Hunan, China; ^6^ Hunan Provincial Clinical Medicine Research Center for Intelligent Management of Chronic Disease, Changsha, Hunan, China

**Keywords:** thyroid nodule, machine learning, early screening, urine iodine, ensemble learning methods

## Abstract

**Background:**

Thyroid nodules, increasingly prevalent globally, pose a risk of malignant transformation. Early screening is crucial for management, yet current models focus mainly on ultrasound features. This study explores machine learning for screening using demographic and biochemical indicators.

**Methods:**

Analyzing data from 6,102 individuals and 61 variables, we identified 17 key variables to construct models using six machine learning classifiers: Logistic Regression, SVM, Multilayer Perceptron, Random Forest, XGBoost, and LightGBM. Performance was evaluated by accuracy, precision, recall, F1 score, specificity, kappa statistic, and AUC, with internal and external validations assessing generalizability. Shapley values determined feature importance, and Decision Curve Analysis evaluated clinical benefits.

**Results:**

Random Forest showed the highest internal validation accuracy (78.3%) and AUC (89.1%). LightGBM demonstrated robust external validation performance. Key factors included age, gender, and urinary iodine levels, with significant clinical benefits at various thresholds. Clinical benefits were observed across various risk thresholds, particularly in ensemble models.

**Conclusion:**

Machine learning, particularly ensemble methods, accurately predicts thyroid nodule presence using demographic and biochemical data. This cost-effective strategy offers valuable insights for thyroid health management, aiding in early detection and potentially improving clinical outcomes. These findings enhance our understanding of the key predictors of thyroid nodules and underscore the potential of machine learning in public health applications for early disease screening and prevention.

## 1 Introduction

Thyroid nodules, as a common clinical condition, have seen a significant increase in detection rates with the widespread use of high-resolution imaging technologies such as ultrasound. Epidemiological studies indicate that the prevalence of thyroid nodules is continuously rising worldwide, with an adult prevalence rate of 33% to 68% (1, 2). Approximately 10%-15% of thyroid nodules may become malignant and develop into thyroid cancer (3). Between 2000 and 2018, the age-standardized incidence rate of cancer in Chinese women increased significantly by 2.6% per year, with thyroid cancer being a major contributing factor (AAPC = 15.7%). By 2022, thyroid cancer had become the third most common new cancer case among all cancers (4). However, due to improvements in early screening and diagnosis, the detection rate of early thyroid nodules is high, leading to a relatively good overall prognosis for thyroid cancer (5).

With the rapid advancement of computational technology, machine learning has emerged as a key technique for handling large datasets and parsing high-dimensional information, overcoming the limitations of traditional statistical methods in processing such data. The core advantage of machine learning over traditional statistics lies in its ability to autonomously explore and learn complex patterns hidden within data, a process that does not depend on predefined model assumptions. By iteratively learning from and optimizing with a large volume of data, machine learning improves the model’s generalization capability for unseen data, making it effectively adaptable to complex real-world problems. Machine learning has already been efficiently applied in medical auxiliary diagnosis for various diseases, including the discrimination and prediction of thyroid nodules and thyroid cancer: Peng et al. (6) developed the ThyNet model, which, through training on ultrasound images, achieved an AUC value of 0.922 for the diagnosis of thyroid tumors on the ROC curve, significantly higher than the 0.839 achieved by radiologists, and the assistance strategy based on this method significantly enhanced the diagnostic capability of radiologists. Jin et al. (7) constructed the Thy-Wise model using demographic data, thyroid function indicators, and thyroid ultrasound features. This model effectively improves the diagnostic accuracy and specificity for assessing thyroid nodules compared to the earlier ACR TI-RADS, and reduces unnecessary thyroid fine needle aspiration biopsies. Yao et al. (8) developed a multimodal deep learning model, DeepThy-Net, based on over 23,000 thyroid ultrasound images and clinical indicators from multiple centers. This model demonstrated good clinical applicability in predicting various types of cervical lymph node metastasis in papillary thyroid carcinoma (AUC 0.870–0.905). Additionally, the team constructed a diagnostic model for Bethesda IV thyroid nodules based on the Transformer architecture, which also showed significant diagnostic value (9).

Due to the high prevalence of thyroid nodules and the potential of some nodules to develop into thyroid cancer, early screening and risk assessment of thyroid nodules are essential for devising appropriate management strategies. Although models built on ultrasound images have shown strong capabilities in identifying thyroid nodules, the human and material costs associated with widespread screening are high and may lead to overdiagnosis and overtreatment. In contrast, utilizing demographic data and routine clinical indicators as features for machine learning models offers the advantages of convenient data collection, good retrospective capabilities, and no additional examination costs. Therefore, the primary objective of this study is to construct a suitable and highly accurate machine learning model based on these indicators to predict the occurrence of thyroid nodules, thereby addressing a current gap in research.

## 2 Materials and methods

### 2.1 Study data

**Figure 1:**
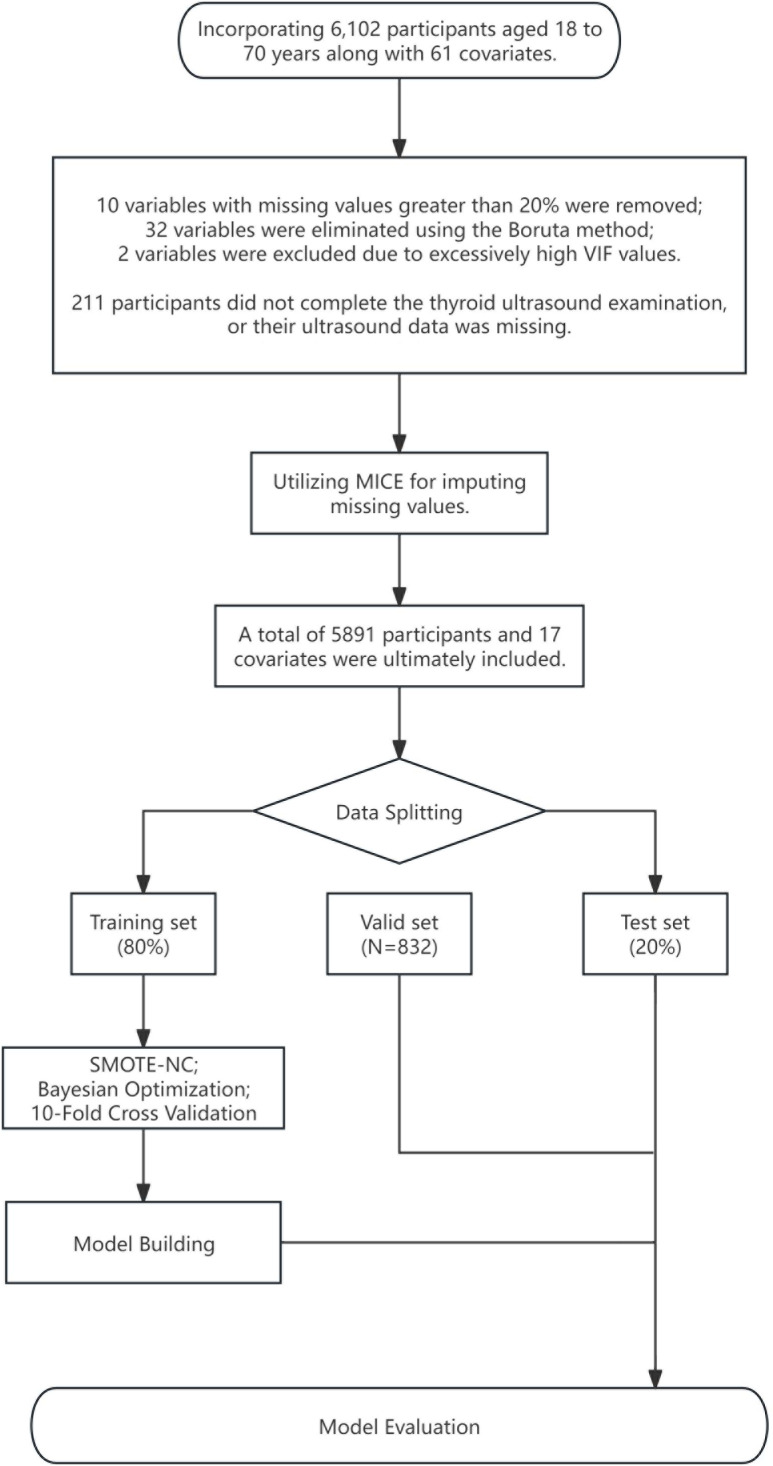
Data processing flowchart. MICE, multiple imputation by chained equations; SMOTE-NC, synthetic minority over-sampling technique for nominal continuous.

The data for this study were sourced from the Health Management Center of Xiangya Hospital Second Affiliated to Central South University in Changsha, China, covering 6,102 participants (**Figure 1**), with an age range of 18 to 70 years old, all without a history of thyroid surgery, collected from January 2022 to December 2023. To protect privacy, no sensitive data were included in the collected information. The dataset contains 61 variables, including demographic data, routine biochemical indices, and thyroid ultrasound data, with the data collection span for each sample maintained within one month. To ensure data integrity, samples with more than 20% missing data in clinical indicators (a total of 10) or incomplete thyroid ultrasound data (a total of 211 cases) were excluded. The remaining data with missing values were imputed using the Multiple Imputation by Chained Equations (MICE) method, with detailed imputation information available in **Supplementary Figure 1**. The external validation cohort consisted of 832 patients collected from 2022 to 2023 by the Xinqiao Hospital in Chongqing, China, whose thyroid ultrasound data were complete, and clinical information was consistent with the training and testing sets. The assessment of thyroid nodules followed the Chinese-Thyroid Imaging Reporting and Data System (C-TIRADS) (10), where TIRADS 1 indicates no thyroid nodules, and TIRADS 2 and above are considered to have thyroid nodules. Thyroid ultrasound examinations at all participating centers were performed by two attending physicians using high-resolution ultrasound equipment with a 5–15 MHz linear array transducer according to a standard protocol, and in case of disagreement, a third expert physician reviewed to ensure the accuracy of the results.

### 2.2 Data processing

The feature selection process is an extremely important step in the construction of machine learning models, as it identifies the most relevant subset of features that significantly enhance classification accuracy and reduces the model’s overfitting. We ultimately included 17 variables for model construction, with the detailed feature selection process available in **Supplementary Table 1**. The choice of these variables depended on the following factors: firstly, the indicators have been proven to have a clear association with thyroid function. Secondly, the inclusion of supplemental variables aims to enhance feature diversity and ensure as many potential variables related to thyroid nodules are covered as possible, utilizing the Boruta method (11) to select features deemed important or tentative. The Boruta method is a feature selection technique based on random forests, designed to identify all relevant features associated with the response variable. It creates “shadow features” by duplicating each feature in the dataset and shuffling these duplicated features’ values to disrupt their original association with the response variable. Then, Boruta uses this extended dataset, containing both original and shadow features, to train a random forest model and assess the importance of each feature. By comparing the importance of the original features with the maximum importance of their corresponding shadow features, Boruta determines whether the original features are significantly important. This process is repeated through multiple iterations, removing features considered “unimportant” after each round until a preset number of iterations is reached. Boruta’s mechanism ensures a comprehensive and thorough feature selection process, helping to capture and provide all features possibly relevant to the response variable. We used the create Data Partition function from the caret package to divide the dataset into training and testing sets, with the testing set comprising 80% of all data. For models sensitive to variable scales, we normalized numerical variables in the dataset before constructing the model and performed one-hot encoding for categorical variables in all models.

In the field of machine learning, class imbalance is a common phenomenon where the number of samples in one class significantly exceeds those in other classes. This imbalance has a notable impact on the training of machine learning models, and the extent of the impact depends on the relative proportions of sample sizes between classes. Therefore, we employ the Synthetic Minority Over-sampling Technique for Nominal and Continuous data (SMOTE-NC) (12) technique to address imbalanced data. SMOTE-NC is an extension of the Synthetic Minority Over-sampling Technique (SMOTE) characterized by its ability to handle both categorical and continuous features. By oversampling the minority class samples and generating synthetic samples through interpolation of existing samples, the SMOTE-NC algorithm achieves a balance in the number of samples within an imbalanced dataset while ensuring that the synthetic samples realistically reflect the characteristics of the original data distribution.

### 2.3 Model construction and evaluation

We selected six common classifiers (13–17) for constructing the machine learning model to predict thyroid nodules: Logistic Regression (LR), Support Vector Machine (SVM), Multilayer Perceptron (MLP), Random Forest (RF), eXtreme Gradient Boosting (XGBoost), and Light Gradient Boosting Machine (LightGBM) models. Before establishing each final machine learning model, we used Bayesian optimization to tune the hyperparameters of the machine learning models. Each parameter combination was subjected to ten-fold cross-validation, selecting the hyperparameter set with the best performance (using the smallest log loss as the benchmark for this study). Subsequently, through comprehensive retraining on the entire training set, the final machine learning model was determined. All optimal parameters and detailed information about the equipment and model building environment can be found in **Supplementary Table 2**.

Following the construction of our machine learning models, we evaluated them by constructing Receiver Operating Characteristic (ROC) curves and comprehensively assessing key performance metrics on both the test set and external validation set, including Accuracy, Precision, Recall, F1 Score, Specificity, Kappa statistic, and the Area Under the Curve (AUC). Furthermore, we employed the DeLong test to statistically compare the performance of all models between the internal validation set and external validation set, assessing the models’ generalizability and stability. Additionally, Decision Curve Analysis (DCA) (18) was utilized to observe the net benefit of different models at various threshold settings for the prediction of thyroid nodules, thereby further guiding the optimization of clinical decision-making. This suite of evaluation methodologies ensures the rigor of our study and the practical utility of our models, providing a scientific basis for the diagnosis of thyroid nodules.

## 3 Results

### 3.1 Characteristics and distribution of participants

**Figure 2:**
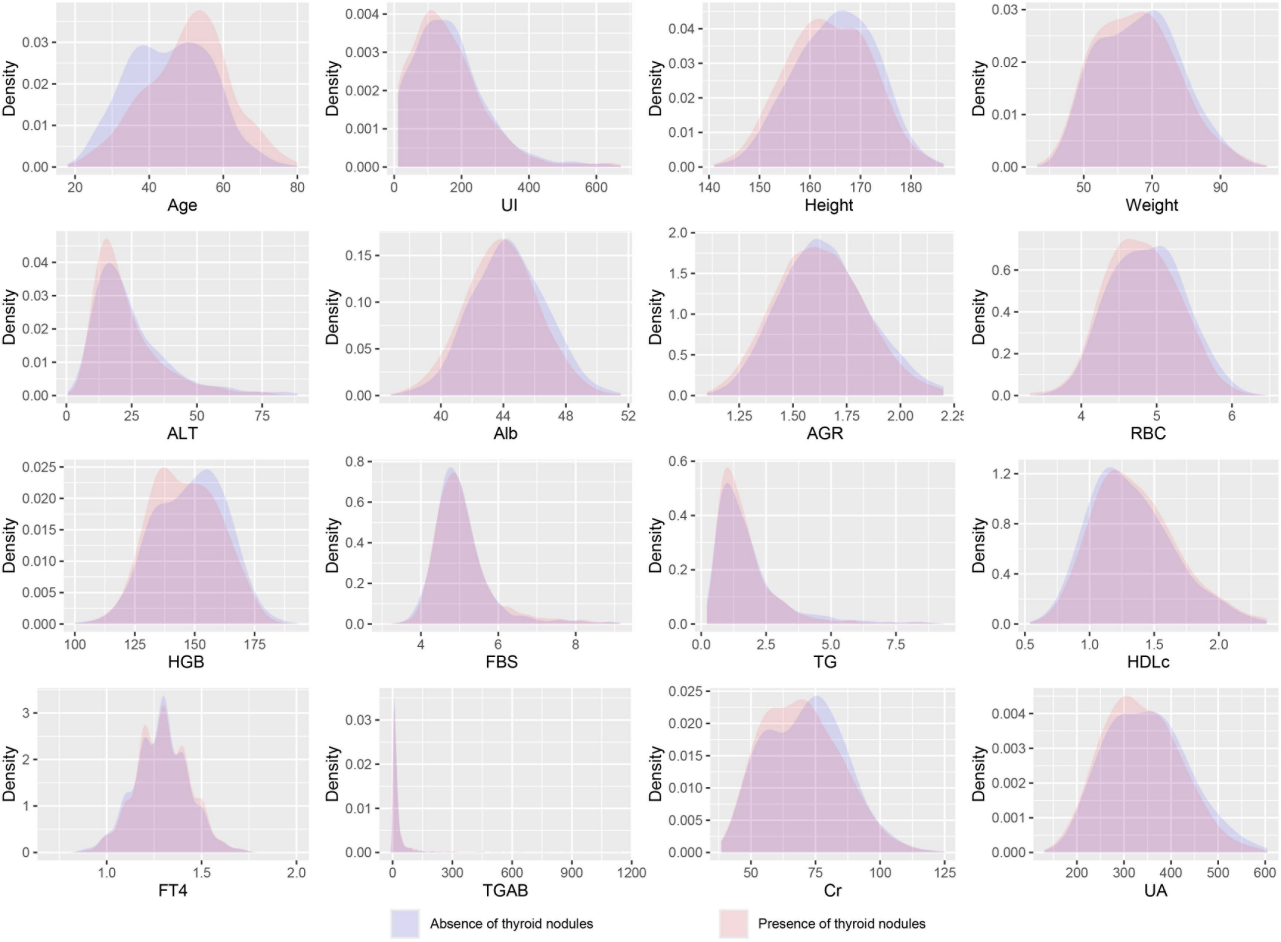
Density distribution curves of all variables. UI, Urine Iodine; ALT, Alanine Aminotransferase; Alb, Albumin; AGR, Albumin/Globulin Ratio; RBC, Red Blood Cells; HGB, Hemoglobin; FBS, Fasting Blood Sugar; TG, Triglycerides; HDLc, High-Density Lipoprotein Cholesterol; FT4, Free Thyroxine; TGAB, Thyroglobulin Antibodies; Cr, Creatinine; UA, Uric Acid.

This study ultimately included 5,891 participants, of which 3,461 were males and 2,430 were females. Among the participants, 2,960 individuals were diagnosed with thyroid nodules. Their average age was 50.6 ± 11.2 years, while the average age of participants without thyroid nodules was 45.7 ± 11.0 years. All included variables are presented as mean (SD) or N (Proportion), and the differences between numeric variables in the two groups were analyzed using the two-sample t-test; the Chi-square test was used to examine the distribution differences of categorical variables (**Table 1**), and the density distribution of all numeric variables can be seen in **Figure 2**. Information on the baseline situation of the external validation dataset can be found in **Supplementary Table 3**.

**Table 1:** Characteristics of study population.

Variables		Absence of thyroid nodules (N=2931)		Presence of thyroid nodules (N=2960)		P-value
		Mean/N	SD/Proportion	Mean/N	SD/Proportion	
Age (year)		45.7	11.0	50.6	11.2	<0.001
Gender						<0.001
Male		1857	63.4%	1604	54.2%	
Female		1074	36.6%	1356	45.8%	
UI (μg/L)		165.9	110.6	158.4	111.3	0.01
Height (cm)		165.1	8.0	163.8	8.1	<0.001
Weight (kg)		66.5	11.8	65.4	11.8	<0.001
ALT (U/L)		24.7	14.8	23.0	13.3	<0.001
Alb (g/L)		44.4	2.4	43.9	2.3	<0.001
AGR		1.7	0.2	1.6	0.2	<0.001
RBC (×10^12/L)		4.9	0.5	4.8	0.5	<0.001
HGB (g/L)		148.3	14.6	146.0	14.1	<0.001
FBS (mmol/L)		5.0	0.8	5.1	0.8	0.004
TG (mmol/L)		1.8	1.4	1.7	1.2	<0.001
HDLc (mmol/L)		1.3	0.3	1.4	0.3	<0.001
FT4 (ng/dL)		1.3	0.1	1.3	0.1	0.048
TGAB (ng/dL)		44.3	112.2	48.0	120.7	0.212
Cr (μmol/L)		71.1	15.1	69.6	15.0	<0.001
UA (μmol/L)		348.6	86.1	337.4	81.6	<0.001

UI, Urine Iodine; ALT, Alanine Aminotransferase; Alb, Albumin; AGR, Albumin/Globulin Ratio; RBC, Red Blood Cells; HGB, Hemoglobin; FBS, Fasting Blood Sugar; TG, Triglycerides; HDLc, High-Density Lipoprotein Cholesterol; FT4, Free Thyroxine; TGAB, Thyroglobulin Antibodies; Cr, Creatinine; UA, Uric Acid. Data are represented as mean (SD) or number (proportion), and the p-values are calculated using the Welch Two Sample t-test or Fisher’s exact test.

### 3.2 Model performance

**Figure 3:**
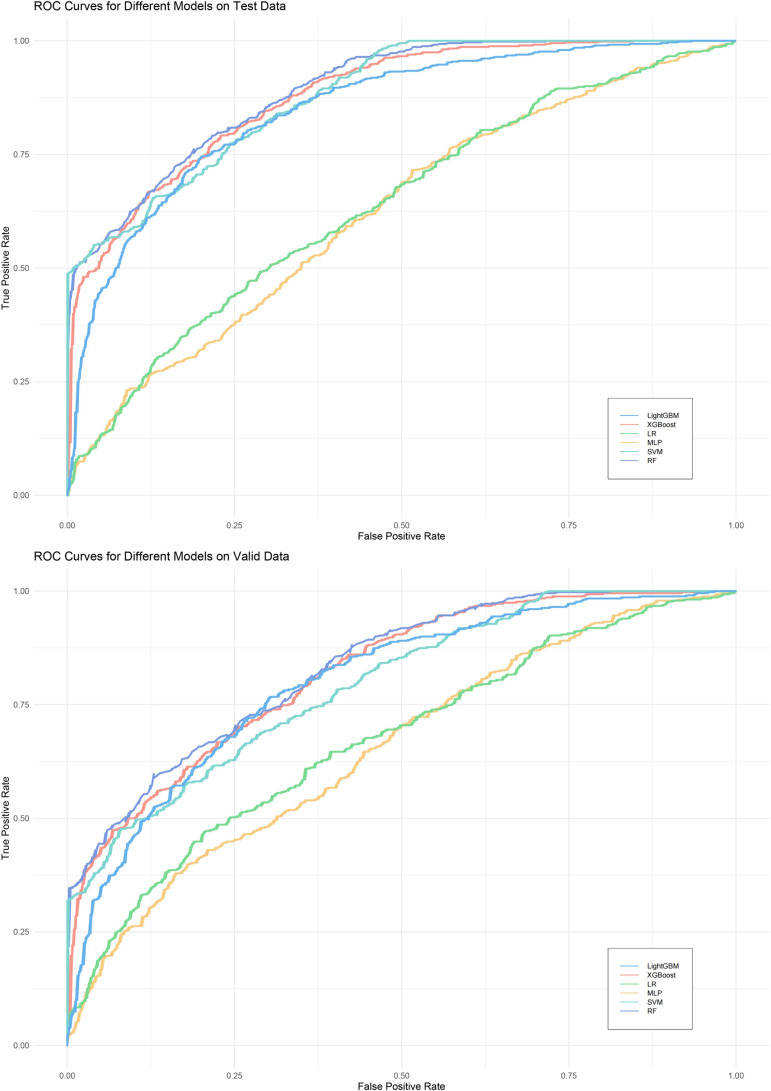
ROC curves of all models. LightGBM, light gradient boosting machine; XGBoost, extreme gradient boosting; LR, logistic regression; MLP, Multilayer Perceptron; SVM, support vector machine; RF, random forest.

**Figure 4:**
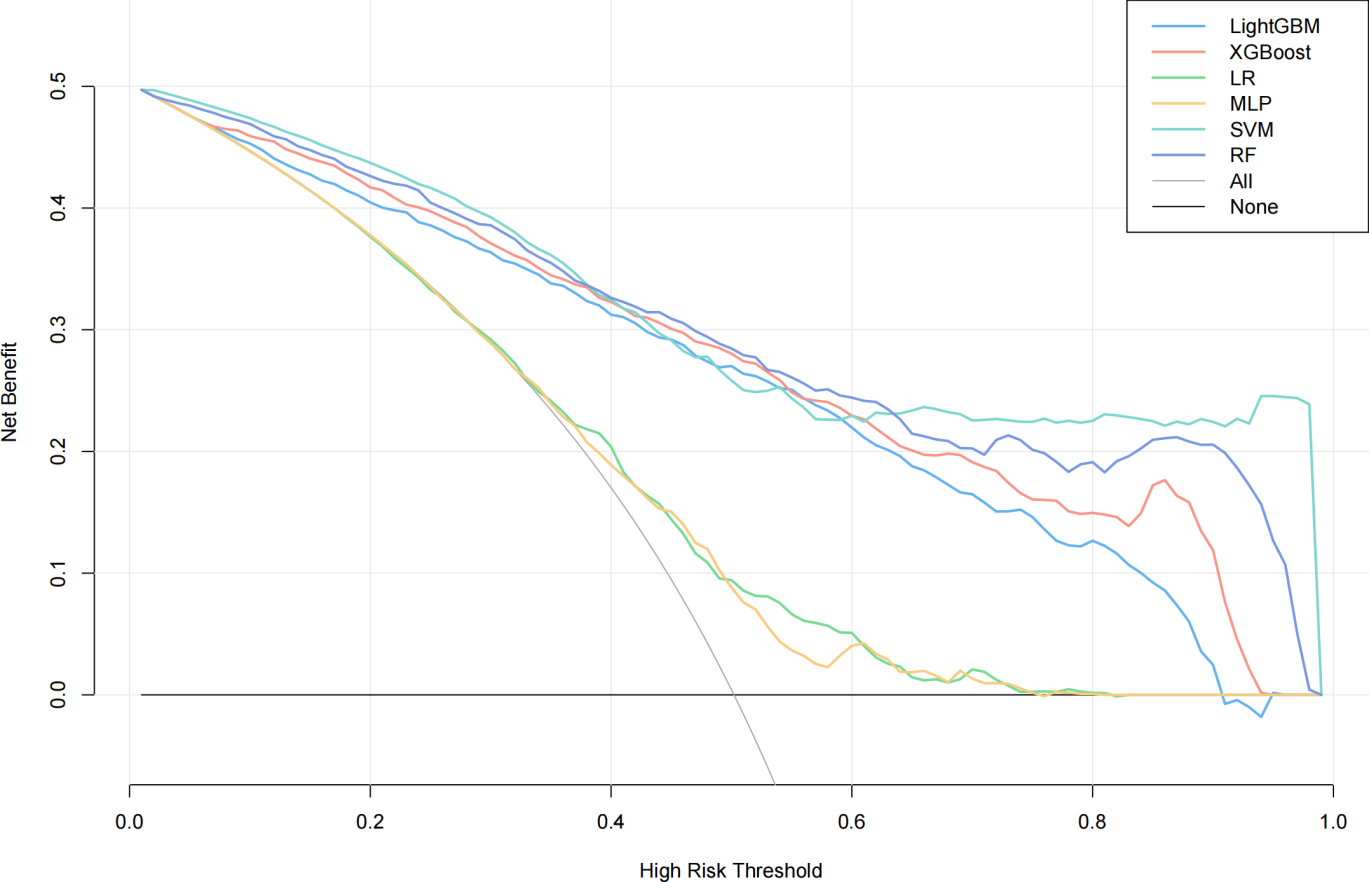
DCA curves of all models. LightGBM, light gradient boosting machine; XGBoost, extreme gradient boosting; LR, logistic regression; MLP, multilayer perceptron; SVM, support vector machine; RF, random forest.

**Figure 5:**
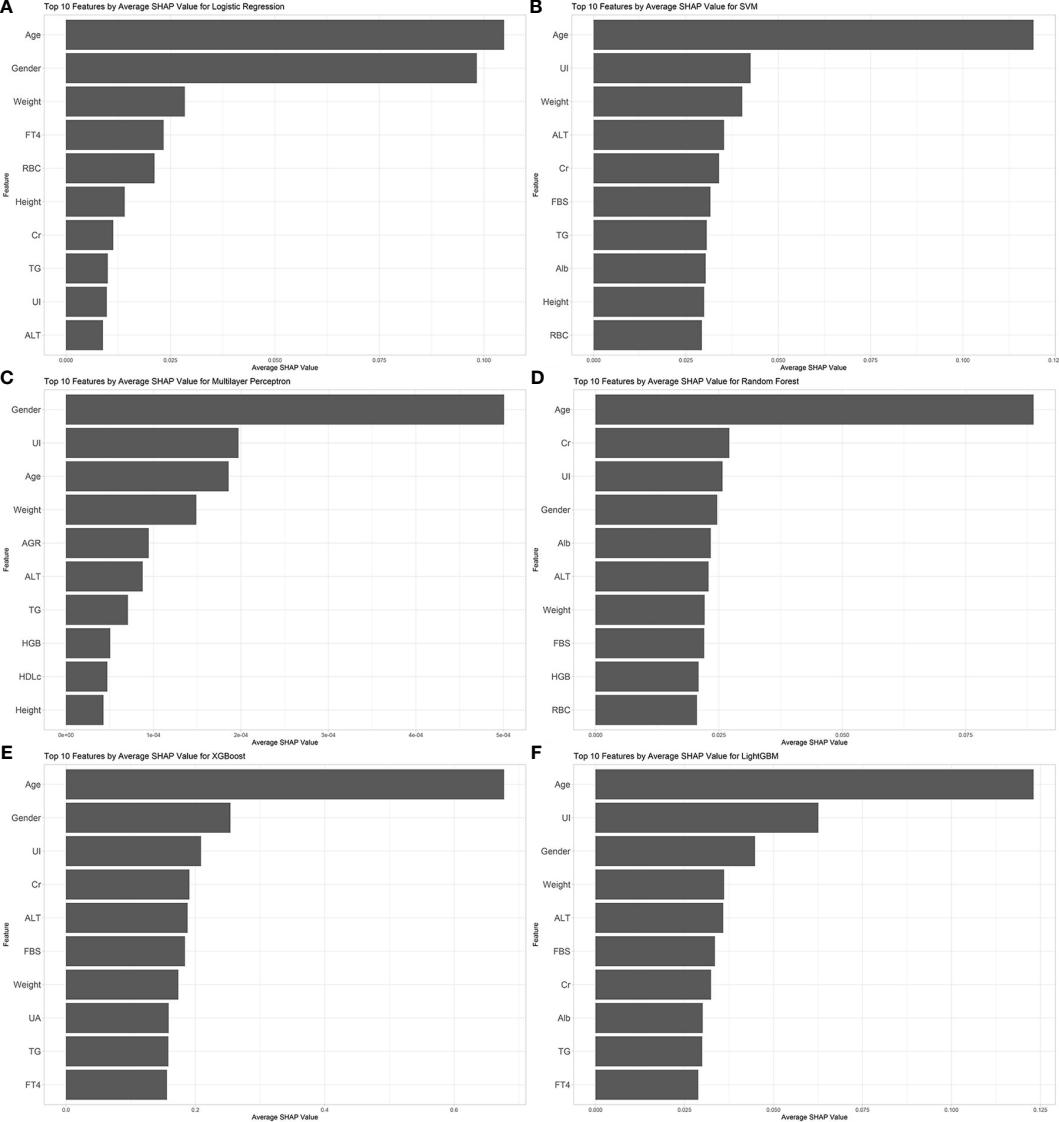
Feature importance analysis of all models. **(A)** logistic regression; **(B)** support vector machine; **(C)** multilayer perceptron; **(D)** random forest; **(E)** extreme gradient boosting; **(F)** light gradient boosting machine. UI, Urine Iodine; ALT, Alanine Aminotransferase; Alb, Albumin; AGR, Albumin/Globulin Ratio; RBC, Red Blood Cells; HGB, Hemoglobin; FBS, Fasting Blood Sugar; TG, Triglycerides; HDLc, High-Density Lipoprotein Cholesterol; FT4, Free Thyroxine; Cr, Creatinine; UA, Uric Acid.

We evaluated the performance of machine learning by constructing ROC curves for various models (**Figure 3**) and calculating different metrics (**Table 2**). In the validation on internal datasets, we found that the Random Forest model had the highest accuracy rate, reaching 78.3%, and possessed the highest AUC value of 89.1%. XGBoost closely followed the Random Forest model in terms of prediction accuracy, with an accuracy rate of 78.0% and an AUC curve also reaching 87.8%, suggesting that the XGBoost model is also a superior prediction model. When the models were applied to external validation data, according to the results of the Delong test, the AUCs of the SVM, Random Forest model, and XGBoost model showed a significant decline compared to the internal test data, while the AUC of LightGBM showed a downward trend but without significant difference, indicating that the LightGBM model still maintains good robustness in external validation. By constructing DCA curves (**Figure 4**), we found that at lower high-risk thresholds, all models had a net benefit for treatment decisions that was higher than taking no action. When the threshold was in the medium range of 0.2–0.8, most models (SVM, LightGBM, XGBoost, Random Forest) showed a higher net benefit, providing valuable information for doctors and assisting them in making decisions. At higher thresholds, the net benefit curves of the SVM, LightGBM, XGBoost, and Random Forest models varied significantly, indicating unstable benefits brought by the models. From the results of feature importance analysis (**Figure 5**), it is not difficult to see that age, gender, and urine iodine levels play an important role in most models.

**Table 2:** Assessment of all machine learning models.

Models	Dataset	Accuracy	Precision	Recall	F1 Score	Specificity	Kappa	AUC	P-value
LR	Test data	0.5667	0.2487	0.6901	0.3657	0.5394	0.1357	0.6391	0.188
	Valid data	0.5889	0.2930	0.7683	0.4242	0.5449	0.1943	0.6712	
MLP	Test data	0.5854	0.6379	0.5791	0.6071	0.5932	0.1704	0.6233	0.22
	Valid data	0.6010	0.6419	0.6079	0.6244	0.5926	0.1995	0.6537	
SVM	Test data	0.7604	0.7597	0.7623	0.7610	0.7585	0.6811	0.8788	< 0.001
	Valid data	0.6947	0.6744	0.7178	0.6954	0.6729	0.5937	0.7946	
RF	Test data	0.7825	0.8003	0.7741	0.7870	0.7915	0.5649	0.8906	< 0.001
	Valid data	0.7175	0.7372	0.7221	0.7296	0.7125	0.4340	0.8322	
XGBoost	Test data	0.7799	0.7902	0.7757	0.7829	0.7843	0.5599	0.8775	< 0.001
	Valid data	0.7188	0.7326	0.7258	0.7292	0.7111	0.4367	0.8199	
LightGBM	Test data	0.7664	0.7665	0.7678	0.7671	0.5327	0.7649	0.8485	0.08
	Valid data	0.7272	0.7488	0.7302	0.7394	0.7238	0.4532	0.7986	

LR, logistic regression; MLP, multilayer perceptron; SVM, support vector machine; RF, random forest; XGBoost, extreme gradient boosting; LightGBM, light gradient boosting machine. The p-value in the table indicates whether there is a significant statistical difference between the AUCs of the ROC curves of the same machine learning model on the internal test set and the external validation set, with a p-value of less than 0.05 considered indicative of a difference.

## 4 Discussion

In this study, through feature engineering of 61 common clinical indicators, we identified 17 key feature variables covering demographic data, blood biochemical indicators, and urine routine test results. We further developed and evaluated six different machine learning models to compare their performance in identifying thyroid nodules. Except for logistic regression and multilayer perceptron models, the accuracy of the other models in internal validation exceeded 76%, with the Random Forest model performing the best followed by the XGBoost model. However, in external validation, according to the results of the Delong test, the LightGBM model showed no significant difference in AUC value compared to internal validation and demonstrated higher robustness with an accuracy rate of 72.72%. The accuracy of the aforementioned three models in external validation all reached over 70%, likely due to their use of ensemble learning methods. These methods can effectively reduce the risk of overfitting while increasing the accuracy and stability of predictions. Moreover, their automatic capture of complex relationships between features and efficient handling of large datasets further enhance the model’s performance in binary classification tasks.

The analysis of SHAP values for the model clearly highlights the importance of age, gender, and urinary iodine as indicators for identifying thyroid nodules. Existing research has shown that compared to younger individuals and males, older individuals and females are at a higher risk of developing thyroid nodules (19, 20). As age increases, the colloid content in thyroid tissue decreases while the interstitial tissue increases (21). Additionally, serum TSH levels gradually rise. This increase in concentration is believed to be related to a decline in the biological activity of TSH, rather than being directly associated with thyroid disease (22). Changes in thyroid hormones and tissue structure may be potential factors leading to the occurrence of thyroid nodules. The incidence of thyroid nodules in women is related to their levels of estrogen and progesterone. Fluctuations in these hormone levels during menopause and reproductive years may promote the growth of thyroid nodules (23). Urinary iodine, an indicator of the body’s iodine metabolism status, is often associated with factors affecting glucose and lipid metabolism (24) as well as thyroid function disorders (25, 26). Studies have indicated a nonlinear U-shaped relationship between urinary iodine and the risk of thyroid nodules, which also exhibits gender differences: the risk of thyroid nodules in males decreases with increasing levels of urinary iodine, whereas the opposite is observed in females (27, 28). Most supporting evidence comes from cross-sectional studies, and further cohort studies or other research capable of explaining causal relationships are needed to verify the relationship between them. In previous models predicting thyroid nodules based on imaging features, the inclusion of thyroid function indicators does not seem to significantly improve the predictive accuracy of machine learning models. Our study’s calculation of Shapley values also confirms this point; however, unlike previous studies, we included urinary iodine as an indicator and highlighted its importance in model construction. This suggests that urinary iodine may play a role akin to a weathervane in the formation of thyroid nodules, warranting further research and exploration.

In the current field of machine learning research, most studies focus on distinguishing between benign and malignant thyroid nodules, with relatively less exploration into the presence or absence of thyroid nodules. These studies typically rely on detailed questionnaires and image feature recognition technologies, which may not be the most critical approach for applications aimed at screening for the presence of thyroid nodules in the so-called “healthy” population. In fact, for this population, our main concern is not the benign or malignant nature of the nodules but their presence, to decide whether further physical examination is needed. Unlike existing research, this paper proposes a new machine learning model that relies solely on common clinical biochemical indicators and demographic information. These data are not only easily obtainable but also relatively low-cost for screening thyroid nodules. Furthermore, by focusing on the presence or absence of thyroid nodules rather than their benignity or malignancy, this study fills a gap in existing research. This approach not only provides a low-cost, efficient solution for the preliminary screening of thyroid nodules but also highlights the potential application of machine learning in the field of public health, especially in early disease identification and prevention.

Despite the valuable insights provided by this study, there are several limitations that need to be acknowledged. First, given the cross-sectional design of this study, we assessed the importance of variables by calculating their importance in the model, yet this approach cannot reveal the causal relationships between variables and thyroid nodules. Further basic research and randomized controlled trials are needed to elucidate these relationships. Second, as a multicenter study, although we standardized the criteria for patient inclusion and the assessment methods for thyroid nodules, the diagnostic outcomes rely on ultrasound examinations, and the use of different operators and ultrasound equipment brands may influence the diagnostic results. Moreover, this study collected demographic data and common clinical examination indicators, but failed to include some factors that may be related to the development of thyroid nodules, such as serum iodine levels, smoking, and drinking habits. Despite these limitations, our study achieved an accuracy of 78% in internal validation and approximately 72% in external validation using the Random Forest model. Future research will focus on collecting these missing features and improving the accuracy of thyroid nodule discrimination through optimizing machine learning models.

## 5 Conclusion

In conclusion, this study effectively harnessed machine learning to predict the presence of thyroid nodules by meticulously selecting 17 key variables from common clinical indicators. Through the development and evaluation of various machine learning models, it was determined that ensemble learning methods, particularly Random Forest and XGBoost, excel in internal validation with high accuracy and robustness, while the LightGBM model demonstrated superior adaptability in external validation. Notably, the analysis underscored the significance of age, gender, and urinary iodine levels as pivotal factors in the identification of thyroid nodules, highlighting the potential of machine learning in filling a critical research gap by focusing on easily accessible clinical data for early screening. This approach not only promises a cost-effective and efficient strategy for preliminary thyroid nodule screening but also opens avenues for public health applications in early disease detection and prevention.

## Data availability statement

The data analyzed in this study is subject to the following licenses/restrictions: The datasets analyzed during the study are not publicly available due to ethical restrictions. The summary information of the data involved in this research has been provided within the main text and the **Supplementary Materials**. Further inquiries about the data and requests for additional details can be directed to the corresponding author. Requests to access these datasets should be directed to pengdq@csu.edu.cn.

## Ethics statement

The studies involving humans were approved by the Ethics Committee of the Second Xiangya Hospital. The studies were conducted in accordance with the local legislation and institutional requirements. Written informed consent for participation was not required from the participants or the participants’ legal guardians/next of kin because Given its cross-sectional design, the requirement for informed consent was waived.

## Author contributions

SW: Writing – original draft, Validation, Software, Methodology. CD: Writing – review & editing. DH: Writing – review & editing, Data curation. JC: Writing – review & editing, Data curation. YL: Writing – review & editing, Data curation. WL: Writing – review & editing. YC: Writing – review & editing, Data curation. XG: Writing – review & editing, Formal Analysis. CC: Writing – review & editing, Investigation. YTY: Writing – review & editing, Visualization. YYY: Writing – review & editing, Supervision, Project administration. DP: Writing – review & editing, Supervision, Resources, Project administration, Funding acquisition.
