# Supplementary material for: Utilizing machine learning for early screening of thyroid nodules: a dual-center cross-sectional study in China
Source: Front Endocrinol (Lausanne). 2024 Jun 14;15:1385167. doi: 10.3389/fendo.2024.1385167 (PMC11211367; doi:10.3389/fendo.2024.1385167)
Supplement: Supplementary file 1 [file DataSheet_1.docx]

Supplementary Material

# Supplementary Figures





**Supplementary Figure 1.** The blue circles or blue curves in the graph represent the original data and the density distribution curve of the original data, respectively. The red circles or red curves represent the imputed data and the density distribution curve of the imputed data, respectively.All of the above are based on the original dataset, with missing data imputed using MICE (Multiple Imputation by Chained Equations) based on predictive mean matching.

AGR: Albumin/Globulin Ratio; Alb: Albumin; ALT: Alanine Aminotransferase; Cr: Creatinine; FBS: Fasting Blood Sugar; FT4: Free Thyroxine; HGB: Hemoglobin; HDLc: High-Density Lipoprotein Cholesterol; RBC: Red Blood Cells; TG: Triglycerides; TGAB: Thyroglobulin Antibodies; UI: Urine Iodine; UA: Uric Acid.


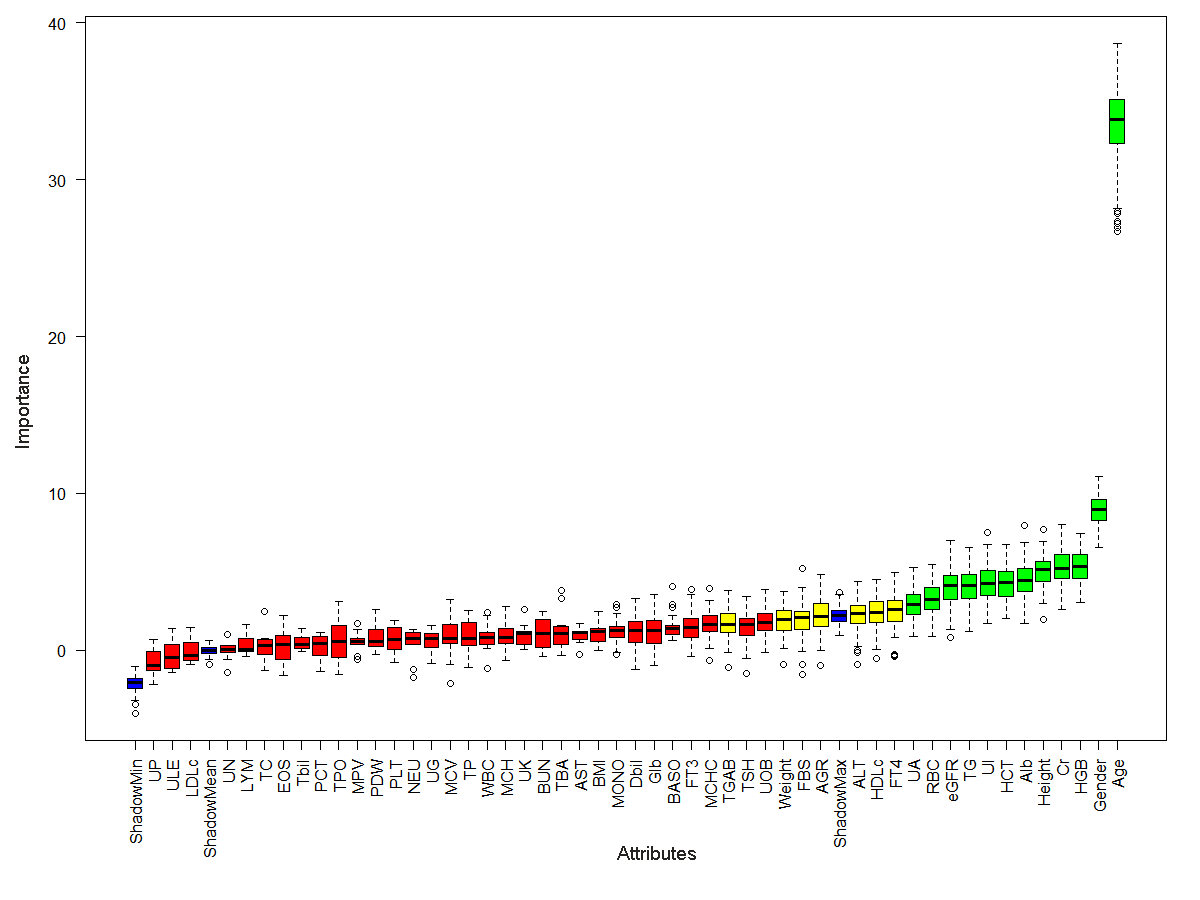


**Supplementary Figure 2.** Feature variable selection using Boruta method.

Different color box plots represent different results of Boruta feature selection: green indicates that the variable was accepted as a feature, yellow suggests the variable is leaning towards acceptance, and red indicates that the variable was rejected. Blue box plots represent shadow variables.

AGR: Albumin/Globulin Ratio; Alb: Albumin; ALT: Alanine Aminotransferase; AST: Aspartate Aminotransferase; BASO: Basophils; BMI: Body Mass Index; BUN: Blood Urea Nitrogen; Cr: Creatinine; Dbil: Direct Bilirubin; EOS: Eosinophils; eGFR: Estimated Glomerular Filtration Rate; FBS: Fasting Blood Sugar; FT3: Free Triiodothyronine; FT4: Free Thyroxine; Glb: Globulin; HCT: Hematocrit; HGB: Hemoglobin; HDLc: High-Density Lipoprotein Cholesterol; LDLc: Low-Density Lipoprotein Cholesterol; LYM: Lymphocytes; MCH: Mean Corpuscular Hemoglobin; MCHC: Mean Corpuscular Hemoglobin Concentration; MCV: Mean Corpuscular Volume; MONO: Monocytes; MPV: Mean Platelet Volume; NEU: Neutrophils; PDW: Platelet Distribution Width; PCT: Procalcitonin; PLT: Platelets; RBC: Red Blood Cells; TBA: Total Bile Acid; TC: Total Cholesterol; TG: Triglycerides; TGAB: Thyroglobulin Antibodies; Tbil: Total Bilirubin; TP: Total Protein; TPO: Thyroid Peroxidase; TSH: Thyroid-Stimulating Hormone; UA: Uric Acid; UG: Urine Glucose; UI: Urine Iodine; UK: Urine Ketones; ULE: Urine Leukocyte Esterase; UN: Urine Nitrite; UOB: Urine Occult Blood; UP: Urine Protein; WBC: White Blood Cells.

# Supplementary Tables

**Supplementary Table 1.** Feature Variable Selection Table.

AGR: Albumin/Globulin Ratio; Alb: Albumin; AG: Anion Gap; ALT: Alanine Aminotransferase; AST: Aspartate Aminotransferase; BASO: Basophils; BMI: Body Mass Index; BUN: Blood Urea Nitrogen; Ca: Calcium; Cl: Chloride; CO2CP: Carbon Dioxide Content/Partial Pressure; Cr: Creatinine; Dbil: Direct Bilirubin; EOS: Eosinophils; eGFR: Estimated Glomerular Filtration Rate; FBS: Fasting Blood Sugar; FT3: Free Triiodothyronine; FT4: Free Thyroxine; Glb: Globulin; HCT: Hematocrit; HGB: Hemoglobin; HDLc: High-Density Lipoprotein Cholesterol; K: Potassium; LDLc: Low-Density Lipoprotein Cholesterol; LYM: Lymphocytes; MCH: Mean Corpuscular Hemoglobin; MCHC: Mean Corpuscular Hemoglobin Concentration; MCV: Mean Corpuscular Volume; Mg: Magnesium; MONO: Monocytes; MPV: Mean Platelet Volume; Na: Sodium; NEU: Neutrophils; P: Phosphorus; PDW: Platelet Distribution Width; PCT: Procalcitonin; PLT: Platelets; RBC: Red Blood Cells; TBA: Total Bile Acid; TC: Total Cholesterol; TG: Triglycerides; TGAB: Thyroglobulin Antibodies; Tbil: Total Bilirubin; TP: Total Protein; TPO: Thyroid Peroxidase; TSH: Thyroid-Stimulating Hormone; UA: Uric Acid; UG: Urine Glucose; UI: Urine Iodine; UK: Urine Ketones; ULE: Urine Leukocyte Esterase; UN: Urine Nitrite; UOB: Urine Occult Blood; UP: Urine Protein; USG: Urine Specific Gravity; WBC: White Blood Cells.

**Supplementary Table 2.** Table for Hyperparameter Selection of All Models.

The table describes various machine learning models that have undergone Bayesian optimization, with each parameter combination subjected to 10-fold cross-validation. Based on the results of the cross-validation, the table displays the set of hyperparameters that achieved the best performance, using the minimum logarithmic loss as the benchmark for this study.

Data preprocessing and model construction were performed using R language (version 4.1.3), Python (version 3.9.7), and TensorFlow (version 2.5.0). The model training process took place on a computer equipped with an NVIDIA RTX 4090 GPU, 64 GB of RAM, and an 11th Gen Intel(R) Core(TM) i5-11400 @ 2.60GHz CPU. The operating system used was Windows 10 Professional 64-bit (Version 21H1 / DirectX 12).

**Supplementary Table 3.** External Validation Data Baseline Table.

AGR: Albumin/Globulin Ratio; Alb: Albumin; ALT: Alanine Aminotransferase; Cr: Creatinine; FBS: Fasting Blood Sugar; FT4: Free Thyroxine; HGB: Hemoglobin; HDLc: High-Density Lipoprotein Cholesterol; RBC: Red Blood Cells; TG: Triglycerides; TGAB: Thyroglobulin Antibodies; UI: Urine Iodine; UA: Uric Acid.
